# Renoprotective effect of a novel combination of 6-gingerol and metformin in high-fat diet/streptozotocin-induced diabetic nephropathy in rats via targeting miRNA-146a, miRNA-223, TLR4/TRAF6/NLRP3 inflammasome pathway and HIF-1α

**DOI:** 10.1186/s40659-024-00527-9

**Published:** 2024-07-20

**Authors:** Merna G. Aboismaiel, Mohamed N. Amin, Laila A. Eissa

**Affiliations:** Department of Biochemistry, Faculty of Pharmacy, Mansoura University, Mansoura, 35516 Egypt

**Keywords:** 6-Gingerol, Diabetic nephropathy, MiRNA-146a, MiRNA-223, NLRP3 inflammasome, TLR4

## Abstract

**Background:**

MiRNA-146a and miRNA-223 are key epigenetic regulators of toll-like receptor 4 (TLR4)/tumor necrosis factor-receptor-associated factor 6 (TRAF6)/NOD-like receptor family pyrin domain-containing 3 (NLRP3) inflammasome pathway, which is involved in diabetic nephropathy (DN) pathogenesis. The currently available oral anti-diabetic treatments have been insufficient to halt DN development and progression. Therefore, this work aimed to assess the renoprotective effect of the natural compound 6-gingerol (GR) either alone or in combination with metformin (MET) in high-fat diet/streptozotocin-induced DN in rats. The proposed molecular mechanisms were also investigated.

**Methods:**

Oral gavage of 6-gingerol (100 mg/kg) and metformin (300 mg/kg) were administered to rats daily for eight weeks. MiRNA-146a, miRNA-223, TLR4, TRAF6, nuclear factor-kappa B (NF-κB) (p65), NLRP3, caspase-1, and hypoxia-inducible factor-1 alpha (HIF-1α) mRNA expressions were measured using real-time PCR. ELISA was used to measure TLR4, TRAF6, NLRP3, caspase-1, tumor necrosis factor-alpha (TNF-α), and interleukin-1-beta (IL-1β) renal tissue levels. Renal tissue histopathology and immunohistochemical examination of fibronectin and NF-κB (p65) were performed.

**Results:**

6-Gingerol treatment significantly reduced kidney tissue damage and fibrosis. 6-Gingerol up-regulated miRNA-146a and miRNA-223 and reduced TLR4, TRAF6, NF-κB (p65), NLRP3, caspase-1, TNF-α, IL-1β, HIF-1α and fibronectin renal expressions. 6-Gingerol improved lipid profile and renal functions, attenuated renal hypertrophy, increased reduced glutathione, and decreased blood glucose and malondialdehyde levels. 6-Gingerol and metformin combination showed superior renoprotective effects than either alone.

**Conclusion:**

6-Gingerol demonstrated a key protective role in DN by induction of miRNA-146a and miRNA-223 expression and inhibition of TLR4/TRAF6/NLRP3 inflammasome signaling. 6-Gingerol, a safe, affordable, and abundant natural compound, holds promise for use as an adjuvant therapy with metformin in diabetic patients to attenuate renal damage and stop the progression of DN.

## Introduction

Diabetes mellitus patients currently experience high rates of morbidity and mortality due to diabetic nephropathy (DN), a serious chronic microvascular complication of diabetes mellitus [1]. DN is the leading cause of chronic kidney disease (CKD) that can progress to end-stage renal disease and is associated with increased cardiovascular mortality [2]. CKD is a broader term that encompasses various conditions resulting in progressive and irreversible kidney damage over time. Aside from DN, other common causes of CKD include hypertensive nephropathy, glomerulonephritis, and polycystic kidney disease [3]. DN is characterized by persistent albuminuria and its pathological features include early podocyte injury, glomerular hyperfiltration, mesangial expansion, glomerular basement membrane thickening, extracellular matrix deposition, glomerulosclerosis, and tubulointerstitial fibrosis [4, 5].

The prevalence of DN is increasing in tandem with the rising prevalence of diabetes mellitus particularly type 2 diabetes mellitus which accounts for about 90% of patients with diabetes [2]. Approximately 20–50% of individuals with type 2 diabetes mellitus will ultimately develop CKD. The prevalence of CKD varies globally, with estimates ranging from 5 to 15% of the population, depending on the region and the criteria used for diagnosis, with DN being responsible for about 50% of cases [6].

In Egypt, the burden of CKD is significant, affecting approximately 13% of the adult population [7]. In 2017, the Global Burden of Disease (GBD) Collaboration estimated that there were 7.1 million individuals with CKD in Egypt, with an age-standardized prevalence of 106 patients with CKD per 1000 population [8]. Similar to the global trend, the burden of CKD has increased by 36% in Egypt, with CKD ranking fifth in leading causes of death from 2009 to 2019 [9]. DN is a leading cause of CKD in Egypt due to the high prevalence of diabetes mellitus in the country, with type 2 diabetes mellitus reporting a prevalence of approximately 16% of all adults aged 20–79 years [10]. According to the Egyptian Renal Data System (ERDS) report conducted in 2020, the most common cause of CKD was hypertension which represented 41% of cases followed by diabetes mellitus which represented 13% of patients with CKD [11].

Toll-like receptor 4 (TLR4)/tumor necrosis factor receptor-associated factor 6 (TRAF6)/NOD-like receptor family pyrin domain-containing 3 (NLRP3) inflammasome signaling pathway is one of the most important inflammatory pathways, which integrates inflammation and fibrosis in diabetes-induced renal injury pathogenesis [12, 13]. TLR4 is activated by pathogen-associated molecular patterns (PAMPs) present on microorganisms or damage-associated molecular patterns (DAMPs) released by damaged or stressed tissues (e.g., ATP) [14]. Activated TLR4 recruits interleukin-1 receptor-associated kinase-4, interleukin-1 receptor-associated kinase-1 (IRAK-1), and TRAF6 to stimulate nuclear factor-kappa B (NF-κB) translocation inside the nucleus and its activation, which triggers transcriptional up-regulation of NLRP3, pro-interleukin-1 beta (IL-1β), and pro-IL-18. This is the priming step, which is not sufficient to immediately promote NLRP3 inflammasome complex assembly and needs an additional activation step [15, 16].

This activation signal involves triggering numerous intracellular events by PAMPs and DAMPs, such as K + efflux, mitochondrial damage, and reactive oxygen species (ROS) generation [17]. Both priming and activation signals trigger NLRP3 inflammasome complex formation, followed by pro-caspase-1 auto-cleavage, which becomes activated and cleaves pro-IL-1β, pro-IL-18, and gasdermin D to induce inflammation and pyroptosis [18].

In addition, NF-κB activation in diabetic kidneys also increases hypoxia-inducible factor-1 alpha (HIF-1α) expression. HIF-1α is the principal regulator of metabolic responses under hypoxic conditions, which are among the earliest incidents of DN development. HIF-1α promotes renal fibrosis, perpetuating a cycle of inflammation and fibrosis in the diabetic kidneys [19–21].

MicroRNAs (miRNAs) are small, non-coding RNA molecules that can bind to targeted mRNAs 3′ untranslated regions (3′ UTR) and induce their degradation or translational repression [22]. Recent studies have highlighted the critical roles of miRNAs as epigenetic regulators of numerous pathways involved in the process of DN development [23–25]. MiRNA-146a is well-known to regulate TLR4-mediated NF-κB activation through a negative feedback mechanism in which NF-κB up-regulates miRNA-146a gene expression while miRNA-146a down-regulates its direct targets, IRAK-1 and TRAF6, downstream of TLR4 signaling to suppress the activity of NF-κB [26, 27] while miRNA-223 exerts a direct inhibitory effect on NLRP3 inflammasome [28].

Metformin (MET) is a glucose-lowering agent that is used as a first-line therapy for type 2 diabetes mellitus. It has been shown to slow the progression of kidney dysfunction through different mechanisms including reducing inflammation, oxidative stress, and fibrosis [29–31]. Despite current treatment strategies, patients continue to develop CKD and end-stage renal disease [32]. Also, oral anti-hyperglycemic drugs have been linked to multiple adverse effects including metformin-associated lactic acidosis and gastrointestinal disturbances [33]. Therefore, the introduction of new therapeutic modalities to protect against the development and progression of DN has become mandatory.

Recently, much attention has been paid to the utilization of compounds from natural sources in the management of different conditions due to their safety, efficacy, and low cost [34–36]. 6-Gingerol (GR) is the major bioactive component of fresh ginger, the rhizome of *Zingiber officinale*, which is one of the most commonly used spices worldwide [37]. 6-Gingerol is a phenolic compound that possesses interesting antioxidant, anti-inflammatory, anticancer, anti-hyperglycemic, and lipid-lowering effects [34–36]. 6-Gingerol was shown to attenuate myocardial fibrosis by reducing oxidative stress, inflammation, and apoptosis through inhibition of the toll-like receptor 4/mitogen-activated protein kinase/nuclear factor-kappa B pathway [38]. Also, 6-gingerol was demonstrated to alleviate pain, anxio-depression, and neuroinflammation in rats with diabetic neuropathy [39]. 6-Gingerol ameliorated weight gain and insulin resistance in metabolic syndrome rats by regulating adipocytokines [40]. 6-Gingerol has also demonstrated a key protective role in DN by regulating oxidative stress and inflammation [41]. In addition, 6-gingerol could suppress transforming growth factor-β1 signaling, thereby inhibiting the activation of fibroblasts and reducing the deposition of extracellular matrix components, which holds promise in mitigating the progression of renal fibrosis in DN [42]. However, the exact mechanism of the renoprotective effect of 6-gingerol has not been completely understood.

A rat model of type 2 diabetes mellitus, developed by Srinivasan et al. in 2005, was used to induce DN through a combination of high-fat diet (HFD) and low-dose (35 mg/kg) streptozotocin (STZ) [43]. The use of a low dose of STZ selectively induces diabetes in high fat-fed rats with insulin resistance, while it fails to induce diabetes in normal rats. This model exhibits stable and persistent hyperglycemia in addition to the lipid abnormalities seen in type 2 diabetes mellitus patients. This model is suitable for studying the pathophysiology of type 2 diabetes mellitus and its complications, including DN, as it involves both insulin resistance and gradual beta-cell dysfunction which resembles the etiology of human type 2 diabetes mellitus.

The objective of the present study was to assess 6-gingerol’s renoprotective effect and its underlying molecular mechanisms in HFD/STZ-induced DN in rats. The hypothesis of targeting miRNA-146a and miRNA-223 and modulation of TLR4/TRAF6/NLRP3 inflammasome pathway was evaluated. Besides, the prospective beneficial effects of using 6-gingerol and metformin in combination were investigated.

## Materials and methods

### Drugs and chemicals

STZ (CAS no.: 18883-66-4), 6-gingerol (CAS no.: 23513-14-6), and metformin (CAS no.: 1115-70-4) were provided by Sigma Aldrich Co., USA. Citric acid and sodium citrate (for preparation of citrate buffer) and carboxymethyl cellulose (CMC) were provided by El-Gomhouria Co., Mansoura, Egypt. Phosphate-buffered saline (PBS) was provided by Biodiagnostic, Giza, Egypt. All the study’s chemicals were of standard analytic grade.

### Animals

This research gained approval from the ethics committee of the Faculty of Pharmacy at Mansoura University in Mansoura, Egypt (Ethical approval no. 2023 − 159). “Principles of Laboratory Animal Care” (National Materials Institute of Health publication No. 85 − 23, revised 1985) were followed in all animal experiments. Adult male Sprague-Dawley rats (200 ± 20 g) were purchased and housed in the animal house of the Faculty of Pharmacy, Mansoura University. Before the experiment began, rats were left for two weeks to acclimatize to standard environmental conditions of temperature (22 ± 2ºC) and lighting (12 h light-dark cycle) with unrestricted access to food and water.

### Induction of type 2 diabetes mellitus

Rats were given high-fat diet (58% fat, 25% protein, and 17% carbohydrate) for four weeks before receiving a single low-dose (35 mg/kg) intraperitoneal (i.p.) injection of STZ following a night fasting. Cold citrate buffer (0.1 M, pH 4.5) was used to freshly prepare STZ [44]. Diabetes induction was confirmed three days following STZ injection via glucometer (Accu-Check Go, Roche Diagnostics, Mannheim, Germany) to measure blood glucose levels from the tail vein. Diabetic rats were defined as those having blood glucose levels ≥ 250 mg/dl. Diabetic rats were continued on high-fat diet until the experiment ended. All treatments were started 3 days following STZ injection and continued for 8 weeks [45].

### Experimental design

**Fig. 1:**
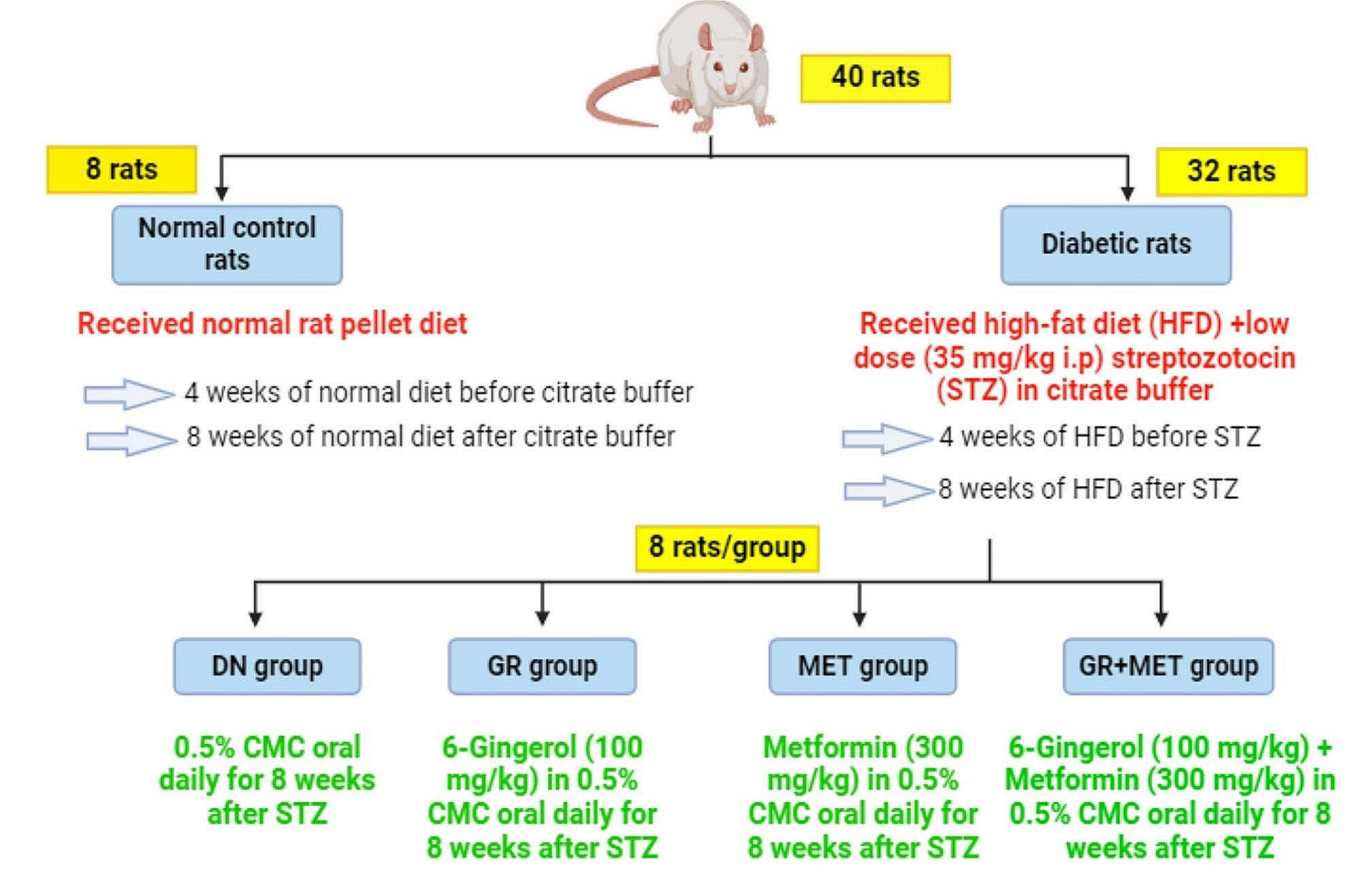
A schematic representation of the experimental design DN: diabetic nephropathy; GR: 6-gingerol; MET: metformin; CMC: carboxymethyl cellulose; HFD: high-fat diet; STZ: streptozotocin

Forty male Sprague-Dawley rats were assigned into five groups (*n* = 8). A schematic representation of the experimental design was described in Fig. 1. The control group, where rats were given a typical rat pellet diet. Then a single i.p. dose of citrate buffer (0.1 M, pH 4.5) was administered to rats after 4 weeks. Three days later, they were administered 0.5% CMC orally every day for 8 weeks. The diabetic nephropathy (DN) group, where untreated diabetic rats received 0.5% CMC orally every day for 8 weeks. The 6-gingerol (GR) group, in which diabetic rats were given an oral dose of 6-gingerol (100 mg/kg) in 0.5% CMC every day for 8 weeks [42]. The metformin (MET) group, in which diabetic rats were given an oral dose of metformin (300 mg/kg) in 0.5% CMC every day for 8 weeks [46]. The 6-gingerol + metformin combination (GR + MET) group, in which diabetic rats received a combination of 6-gingerol (100 mg/kg) and metformin (300 mg/kg) in 0.5% CMC orally every day for 8 weeks.

### Sample collection

At the end of the study, 24-hour urine samples were collected from each rat using metabolic cages (Nalgene, Rochester, NY, USA). Then the rats were fasted overnight and weighed. Thiopental anesthesia (40 mg/kg i.p.) was used to obtain blood samples from the retro-orbital plexus, then blood samples were centrifuged at 3,000 rpm for 15 min at 4 °C to obtain serum samples which were then kept at -20 °C. Then rats were decapitated and sacrificed, after which their kidneys were separated and weighed. Two parts of the left kidney were dissected, frozen instantly with liquid nitrogen, and later kept at -80 °C. The first part was used for biochemical analysis in kidney homogenate, and the second part was employed for quantitative, real-time polymerase chain reaction (qRT-PCR). 10% neutral buffered formalin was used to fix the right kidney to be utilized in histopathology and immunohistochemistry investigations.

### Biochemical analysis in serum

Serum samples were used for measuring fasting blood glucose, serum creatinine, blood urea nitrogen (BUN), triglycerides, total cholesterol, and high-density lipoprotein (HDL) cholesterol concentrations by spectrophotometry using biochemical kits (Biodiagnostic Co., Giza, Egypt) guided by the manufacturer’s recommendations.

### Biochemical analysis in urine

Centrifugation was performed on the collected 24-hour urine samples for 10 min at 4 °C at 2000 rpm. Afterward, urine protein and creatinine concentrations were measured in the resulting supernatants by spectrophotometry technique using commercial biochemical kits (Biodiagnostic Co., Giza, Egypt) as per the manufacturer’s guidelines. Also, urinary protein excretion level (mg/24 hr), creatinine clearance (ml/min), and proteinuria/creatininuria ratio were calculated [45].

### Biochemical analysis in kidney homogenate

First, kidney tissue was rinsed with ice-cold PBS to remove any remaining blood. Then, tissue fragments were weighed and homogenized in PBS (pH 7.4) on ice using a glass homogenizer to prepare 10% (w/v) homogenate. This was followed by sonication for 60 s to further homogenate the cells. Five minutes were spent centrifuging the renal tissue homogenate at 10,000 rpm at 4 °C followed by separation of the supernatant to be employed in biochemical assays [47].

#### Enzyme-linked immunosorbent assay (ELISA)

Commercial ELISA assay kits were used to determine renal concentrations of TLR4 (LifeSpan Biosciences, WA, USA; Catalog No. LS-F4846), TRAF6 (LifeSpan Biosciences, WA, USA; Catalog No. LS-F20212), NLRP3 (Aviva Systems Biology, CA, USA; Catalog No. OKCD04232-48), caspase-1 (Biovision, CA, USA; Catalog No. E4594-100), tumor necrosis factor –alpha (TNF-α) (MyBioSource, CA, USA; Catalog No. MBS2507393), and IL-1β (Abcam, Waltham, MA, USA; Catalog No. ab100768) in kidney homogenate according to the manufacturer’s protocols. The Bradford method (1976) was applied to assess the tissue’s protein content [48] using a Bradford Assay Kit (Abcam, Waltham, MA, USA; Catalog No. ab102535).

#### Assay of oxidative stress and lipid peroxidation biomarkers

Spectrophotometric assay kits from Biodiagnostic Co., Giza, Egypt were utilized to determine renal reduced glutathione (GSH) and malondialdehyde (MDA) levels in renal homogenate as well as serum and urinary MDA levels following the manufacturer’s guidelines.

### Histopathological examination of renal tissue

The formalin-fixed right kidney was dissected longitudinally into two halves before being paraffin-embedded. Renal tissue Sect. (5 μm thick) were stained differently in two slide sets. Hematoxylin and eosin (H&E) staining was applied to the first set to assess renal histopathological alterations [49], which were assessed semi-quantitatively and given scores from 0 to 3, where 0 is normal, 1 is mild, 2 is moderate, and 3 is severe. Masson’s trichrome staining was applied to the second set for assessment of renal interstitial fibrosis as indicated by Yamate, et al. [50]. Images were captured using Nikon digital camera-aided computer software. The percentage of Masson’s-positive area was evaluated quantitatively using Image J analysis software (NIH, USA). The histologist was kept unaware of the experimental groups and a random examination of the slides was carried on.

### Immunohistochemical examination of renal tissue

Renal tissue sections were used for immunohistochemical evaluation of NF-κB (p65) and fibronectin in kidney tissue. Slides were first deparaffinized in xylene, then rehydrated in graded ethanol, and immersed for 10 min at room temperature in a 0.3% H_2_O_2_/methanol solution. The anti-NF-κB (p65) (Catalog No. sc-8008) and anti-fibronectin (Catalog No. sc-8422) from Santa Cruz Biotechnology, USA were 1:100-diluted and incubated overnight on the slides at 4 °C. PBS was employed three times to rinse the slides, followed by incubation for thirty minutes at ambient temperature with anti-rat IgG secondary antibody (Abcam, Waltham, MA, USA; Catalog No. ab150165). The slides were then visualized with diaminobenzidine and finally counterstained with Mayer’s hematoxylin. For the preparation of the negative control procedure, normal rat serum was used instead of the primary antibody. Immunostaining of both NF-κB (p65) and fibronectin showed distinctive brown reactions. Image J analysis software (NIH, USA) was utilized for measuring the area of positive expression to provide a quantitative assessment [51, 52].

### Quantitative, real-time polymerase chain reaction (qRT-PCR)

The miRNeasy Mini Kit (Qiagen, Hilden, Germany; Catalog No. 217,004) was used for the extraction of total RNA, including miRNA, from renal tissue, following the guidelines of the manufacturer. Qiagen’s QuantiTect^®^ Reverse Transcription Kit (Catalog No. 205,311) and miRCURY^®^ LNA^®^ RT Kit (Catalog No. 339,340) were used for synthesizing cDNA from mRNA and miRNA, respectively, guided by the manufacturer’s instructions. PikoReal™ Real-time PCR System (Thermo Fisher Scientific Inc., Waltham, MA, USA) was used for the detection of mRNA and miRNA using Qiagen’s QuantiTect^®^ SYBR^®^ Green RT-PCR Kit (Catalog No. 204,243) and miRCURY^®^ LNA^®^ SYBR^®^ Green PCR Kit (Catalog No. 339,345), respectively, guided by the manufacturer’s instructions. The housekeeping genes for mRNA and miRNA were rat glyceraldehyde-3-phosphate dehydrogenase (GAPDH) and U6, respectively. Based on gene sequences derived from the GenBank, specific primers were designed for TLR4, TRAF6, NF-κB p65, NLRP3, caspase-1, HIF-1α, and GAPDH genes and then analyzed using NetPrimer (PREMIER Biosoft, USA). Table 1 shows a list of the primer sequences. Qiagen’s miRCURY LNA miRNA PCR assays were used to analyze the expression of miRNA. The following primer sets were used: hsa-miR-146a-5p, rno-miR-223-3p, and U6 snRNA. The 2^−ΔΔCT^ method was applied to determine mRNA and miRNA’s relative expression corresponding to GAPDH and U6, respectively.

**Table 1:** Primer sequences of specific genes

Gene of Interest		Primer Sequence	Reference Sequence
TLR4	Forward	5`- CCAGAGCCGTTGGTGTATCT-3`	NM_019178.2
	Reverse	5`- AGAAGATGTGCCTCCCCAGA-3`	
TRAF6	Forward	5`- TCTCCCCTGCCTTCATTGTT − 3`	NM_001107754.2
	Reverse	5`- AGGCTGGCGATTTTGTGTTT − 3`	
NF-κB (p65)	Forward	5`- TGTGTGAAGAAGCGAGACCTG − 3`	NM_199267.2
	Reverse	5`- AAAATCGGATGCGAGAGGAC − 3`	
NLRP3	Forward	5`- GTAGGTGTGGAAGCAGGACT − 3`	NM_001191642.1
	Reverse	5`- CCTTTGCTCCAGACCCTACA − 3`	
Caspase 1	Forward	5`- CGTCTTGCCCTCATTATCTGC − 3`	NM_012762.3
	Reverse	5`- ACAGTATACCCCAGATCCTGC − 3`	
HIF-1α	Forward	5`- GCATCTCCACCTTCTACCCA − 3`	NM_024359.2
	Reverse	5`- TCTGTCTGGTGAGGTTGTCC − 3`	
GAPDH	Forward	5`- CCATCAACGACCCCTTCATT − 3`	NM_017008.4
	Reverse	5`- CACGACATACTCAGCACCAGC − 3`	

TLR4: Toll-like receptor 4, TRAF6: Tumor necrosis factor receptor-associated Factor 6, NF-κB (p65): Nuclear factor-kappa B (p65), NLRP3: NOD-like receptor family pyrin domain-containing 3, HIF-1α: Hypoxia-inducible factor-1 alpha, GAPDH: Glyceraldehyde-3-phosphate dehydrogenase

### Statistical analysis

The results were statistically analyzed using Graph Pad Prism 7 (San Diego, CA, USA). Data normality was tested using Shapiro-Wilk test. One-way analysis of variance (ANOVA) and Tukey’s post-hoc test were employed for the analysis of parametric data, which were presented as mean ± standard error of the mean (SEM). Since statistical analysis for H&E histopathological lesions appeared non-parametric, Kruskal-Wallis and Dunn’s tests were used for the analysis of data, which were presented as median and range. Differences between groups were deemed statistically significant when the *p*-value was below 0.05.

## Results

### 6-Gingerol, Metformin, and their combination attenuated renal injury and improved renal functions in diabetic rats

**Fig. 2:**
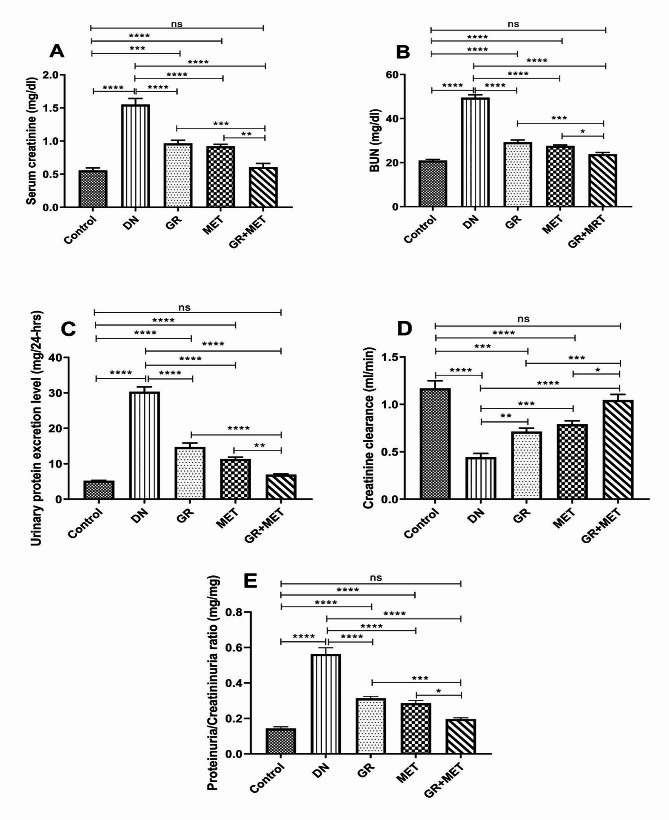
GR, MET, and their combination attenuated renal injury and improved renal functions in diabetic rats Renal functions assessment by measuring serum levels of A: serum creatinine, B: blood urea nitrogen (BUN); C: urinary protein excretion level; D: creatinine clearance; and E: proteinuria/creatininuria ratio. DN: diabetic nephropathy; GR: 6-gingerol; MET: metformin. Data are represented as Mean $$ \pm $$ SEM (*p$$ <$$ 0.05, **p$$ <$$ 0.01, ***p$$ < $$0.001, ****p$$ <$$ 0.0001, ns: non-significant)

Serum creatinine and BUN concentrations, urinary protein excretion level, creatinine clearance, and proteinuria/creatininuria ratio were measured to assess the renoprotective effects of 6-gingerol, metformin, and their combination. As shown in Fig. 2A, B, and C, diabetic rats exhibited a 2.8-fold, 2-fold, and 5.8-fold elevation of serum creatinine, BUN, and urinary protein excretion levels, respectively, corresponding to control rats (*p* < 0.0001). Administration of 6-gingerol, metformin, and their combination to diabetic rats brought about a significantly reduced serum creatinine concentration by 37.7%, 40.6%, and 60.8%, respectively, and BUN by 38.7%, 42.2%, and 49.8%, respectively, while urinary protein excretion levels were reduced by 51.4%, 62.6%, and 77.1%, respectively. Besides, serum creatinine and BUN concentrations, as well as urinary protein excretion levels, were significantly reduced upon treatment of diabetic rats with 6-gingerol + metformin combination as compared to those treated with either 6-gingerol (*p* < 0.001), (*p* < 0.001), (*p* < 0.0001) or metformin (*p* < 0.01), (*p* < 0.05) and (*p* < 0.01), respectively. Compared to the control group, 6-gingerol and metformin groups showed a significant increase in serum creatinine (*p* < 0.0001) and (*p* < 0.001), respectively, and BUN (*p* < 0.0001) concentrations and urinary protein excretion level (*p* < 0.0001), while no significant difference was found in 6-gingerol + metformin group with respect to control group.

On the other hand, Fig. 2D showed that diabetic rats exhibited a 2.5-fold reduction in creatinine clearance relative to the control group (*p* < 0.0001). Creatinine clearance was significantly elevated in 6-gingerol (*p* < 0.01), metformin (*p* < 0.001), and 6-gingerol + metformin combination (*p* < 0.0001) groups relative to the DN group. Besides, creatinine clearance manifested a significant elevation in diabetic rats receiving the 6-gingerol + metformin combination when compared to those treated with 6-gingerol (*p* < 0.001) or metformin (*p* < 0.05) alone. Compared to the control group, 6-gingerol and metformin groups showed a significant decrease in creatinine clearance (*p* < 0.0001) and (*p* < 0.001), respectively, while no significant difference was found in 6-gingerol + metformin group with respect to control group.

Furthermore, Fig. 2E demonstrated a 3.9-fold elevation in proteinuria/creatininuria ratio in diabetic rats with respect to control group (*p* < 0.0001). Proteinuria/creatininuria ratio was markedly reduced in 6-gingerol, metformin, and 6-gingerol + metformin combination groups relative to the DN group (*p* < 0.0001). In addition, proteinuria/creatininuria ratio exhibited a significant reduction in diabetic rats receiving the 6-gingerol + metformin combination when compared to those treated with 6-gingerol (*p* < 0.001) or metformin (*p* < 0.05) alone. Also, proteinuria/creatininuria ratio was significantly elevated in 6-gingerol and metformin groups with respect to control group (*p* < 0.0001) whereas a non-significant difference was observed in 6-gingerol + metformin group relative to the control group. Interestingly, renal functions were markedly improved in rats treated with the 6-gingerol + metformin combination, as indicated by the non-significant variation in serum creatinine and BUN concentrations, urinary protein excretion level, creatinine clearance, and proteinuria/creatinuria ratio with respect to control rats.

**Fig. 3:**
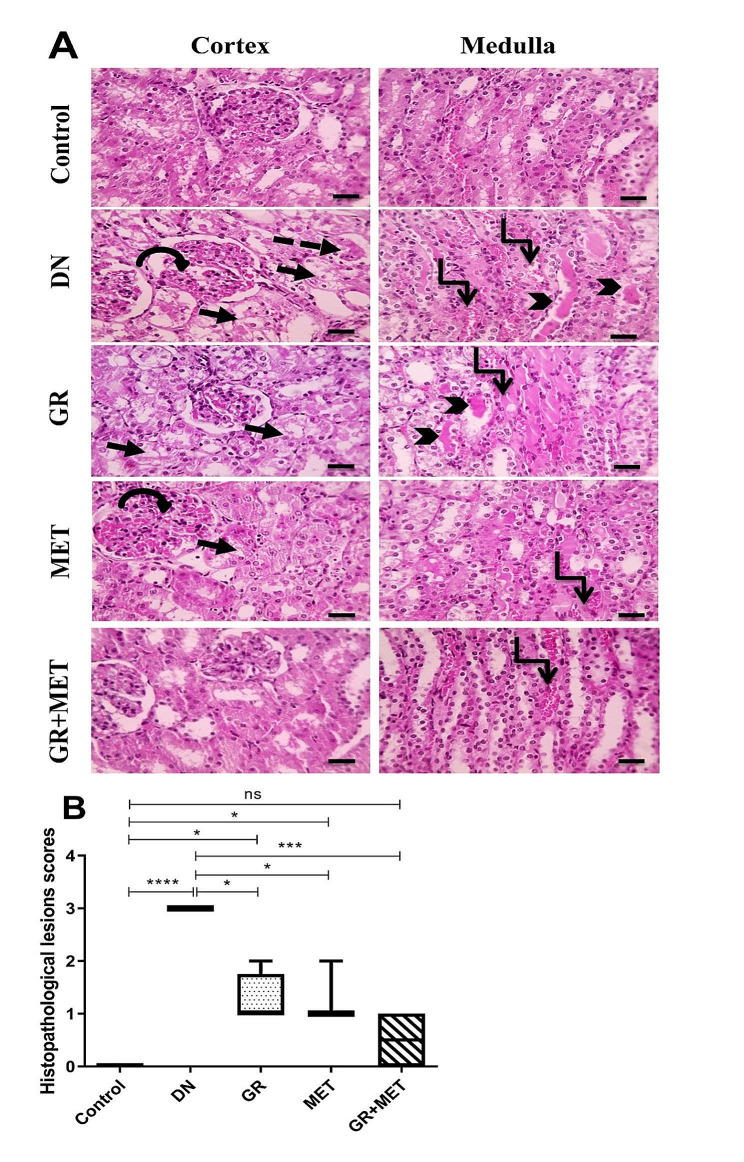
Histopathological examination of H&E-stained renal sections showing renoprotective effects of GR, MET, and their combination **A**: Microscopic images of hematoxylin and eosin (H&E)-stained renal sections showing normal cortex and medulla in the control group, severe pathological changes in the cortex and medulla in DN group, moderately decreased pathological changes in the cortex and medulla in GR group and MET group, and markedly improved histological picture with mild pathological changes in the cortex and medulla in GR + MET group. Black arrows: diffuse tubular hydropic degeneration, dashed arrows: tubular necrosis, black arrowheads: cast formation, circular arrows: congested glomeruli, elbow arrows: congested inter-tubular blood vessels. X: 400, scale bar = 50 micrometer **B**: Renal histopathological changes were assessed semi-quantitatively and given scores from 0 to 3, where 0 is normal, 1 is mild, 2 is moderate, and 3 is severe. DN: diabetic nephropathy, GR: 6-gingerol, MET: metformin. Data are represented as median and range (*p$$ <$$ 0.05, ***p$$ < $$0.001, ****p$$ <$$ 0.0001, ns: non-significant)

The renoprotective effects of the 6-gingerol, metformin, and 6-gingerol + metformin combination were further examined in kidney tissue segments stained with H&E (Fig. 3A and B). Microscopic pictures from the control group showed normal kidney architecture in both the cortex and medulla, with normal glomerular and tubular structures. Severe histopathological lesions were observed in the DN group in both cortex and medulla, including diffuse tubular hydropic degeneration, tubular necrosis, hyaline casts, congested glomeruli, and congested inter-tubular blood vessels. 6-Gingerol group showed moderately decreased histopathological lesions as compared to the DN group (*p* < 0.05) and exhibited moderate tubular hydropic degeneration, hyaline casts, and congested inter-tubular blood vessels. Metformin group showed moderately decreased histopathological lesions compared to the DN group (*p* < 0.05) including moderate tubular hydropic degeneration, congested glomeruli, and mildly congested inter-tubular blood vessels. The 6-gingerol + metformin group showed a significantly improved histopathology relative to DN group (*p* < 0.001) and displayed mild tubular hydropic degeneration and mildly congested inter-tubular blood vessels which seemed non-significantly different from the control group.

### 6-Gingerol, Metformin, and their combination down-regulated TLR4/TRAF6/NLRP3 inflammasome pathway gene expression in DN

**Fig. 4:**
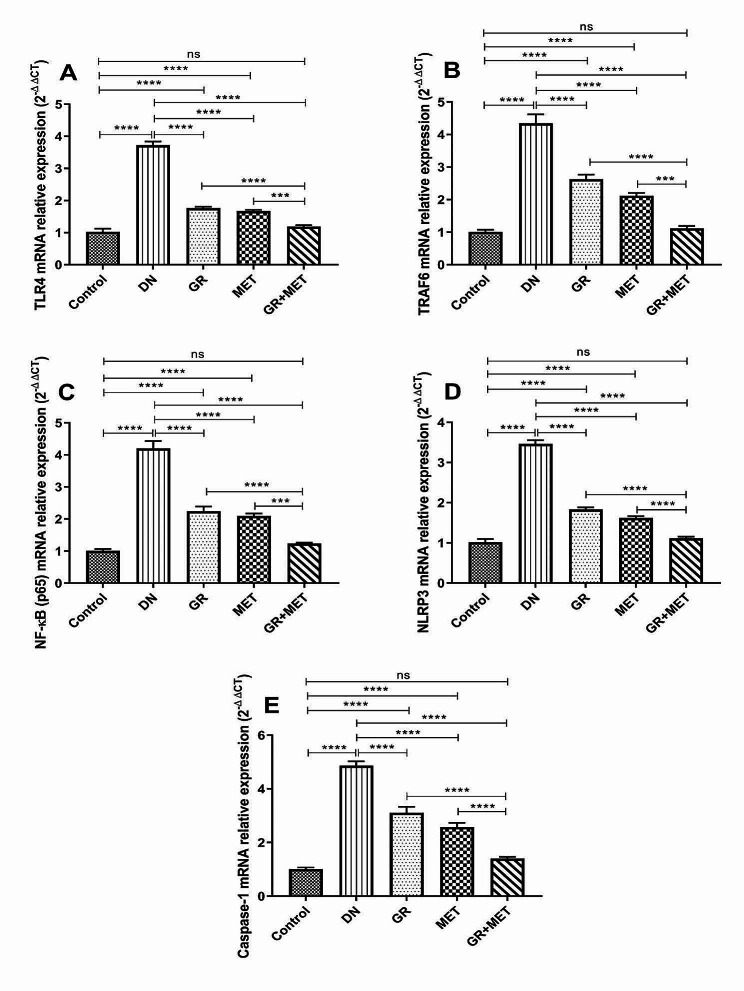
GR, MET, and their combination down-regulated TLR4/TRAF6/NLRP3 inflammasome pathway gene expression in DN Quantitative real-time polymerase chain reaction (qRT-PCR) was used to measure mRNA relative expression of TLR4 (**A**), TRAF6 (**B**), NF-κB (p65) (**C**), NLRP3 (**D**), and caspase-1 (**E**) in renal tissue. DN: diabetic nephropathy; GR: 6-gingerol; MET: metformin; TLR4: Toll-like receptor 4; TRAF6: Tumor necrosis factor receptor-associated Factor 6; NF-κB (p65): Nuclear factor-kappa B (p65); NLRP3: NOD-like receptor family pyrin domain-containing 3. Data are represented as Mean $$ \pm $$ SEM (***p$$ < $$0.001, ****p$$ <$$ 0.0001, ns: non-significant)

As shown in Fig. 4A, B, C, D, and E, the DN group revealed a significantly increased mRNA expression of each of TLR4 (3.6-fold), TRAF6 (4.3-fold), NF-κB (p65) (4.2-fold), NLRP3 (3.4 fold), and caspase-1 (4.8 fold) relative to the control group (*p* < 0.0001). Diabetic rats treated with 6-gingerol, metformin, and 6-gingerol + metformin combination showed significantly lower mRNA expressions of TLR4 by 52.5%, 55%, and 68%, respectively; TRAF6 by 39.5%, 51.1%, and 74.2%; NF-κB (p65) by 46.6%, 50.1%, and 70.5%; NLRP3 by 47.2%, 53.2%, and 67.9%; and caspase-1 by 36.1%, 47%, and 71.2%, respectively.

In addition, the 6-gingerol + metformin group displayed significantly lower TLR4, TRAF6, and NF-κB (p65) mRNA expressions relative to 6-gingerol (*p* < 0.0001) or metformin (*p* < 0.001) group and a significant decrease in NLRP3 and caspase-1 mRNA expressions as compared to 6-gingerol or metformin group (*p* < 0.0001). 6-Gingerol and metformin groups showed a significant increase in TLR4, TRAF6, NF-κB (p65), NLRP3, and caspase-1 mRNA expressions relative to control group (*p* < 0.0001) while no significant variation was seen between 6-gingerol + metformin group and control group.

### 6-Gingerol, Metformin, and their combination suppressed inflammation and pyroptosis in diabetic kidneys

**Fig. 5:**
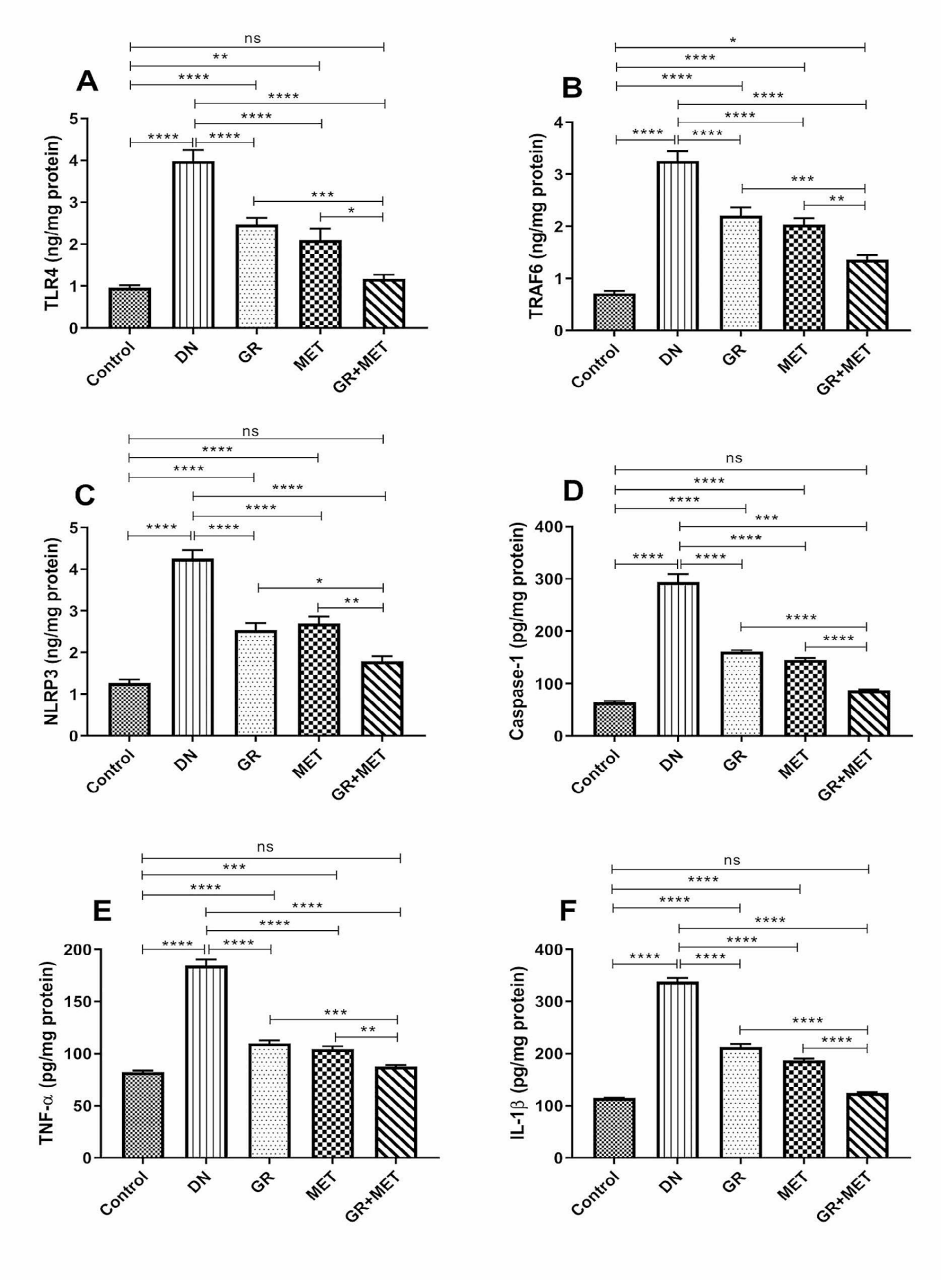
GR, MET, and their combination suppressed inflammation and pyroptosis in diabetic kidneys Renal tissue protein levels of TLR4 (**A**), TRAF6 (**B**), NLRP3 (**C**), caspase-1 (**D**), TNF-α (**E**), and IL-1β (**F**). DN: diabetic nephropathy; GR: 6-gingerol; MET: metformin; TLR4: Toll-like receptor 4; TRAF6: Tumor necrosis factor receptor-associated Factor 6; NLRP3: NOD-like receptor family pyrin domain-containing 3; TNF-α: tumor necrosis factor-alpha; IL-1β: interleukin-1 beta. Data are represented as Mean $$ \pm $$ SEM (*p$$ <$$ 0.05, **p$$ <$$ 0.01, ***p$$ < $$0.001, ****p$$ <$$ 0.0001, ns: non-significant)

In addition to gene expression, TLR4, TRAF6, NF-κB (p65), NLRP3, and caspase-1 protein levels were further determined in renal tissue. As shown in Fig. 5A, B, C, and D, diabetic rats revealed a 4-fold elevation in TLR4, a 4.5-fold elevation in TRAF6, a 3.4-fold elevation in NLRP3, and a 4.6-fold elevation in caspase-1 levels relative to the control group (*p* < 0.0001). Administering 6-gingerol, metformin, or 6-gingerol + metformin combination to diabetic rats brought about significantly reduced levels of TLR4 by 38%, 47.3%, and 70.5%, respectively; TRAF6 by 32.3%, 37.6%, and 58.1%; NLRP3 by 40.4%, 36.6%, and 57.9%; and caspase-1 by 45.2%, 50.5%, and 70.5%, respectively. Furthermore, the 6-gingerol + metformin group revealed significantly reduced levels of TLR4 (*p* < 0.001), (*p* < 0.05); TRAF6 (*p* < 0.001), (*p* < 0.01); NLRP3 (*p* < 0.05), (*p* < 0.01); and caspase-1 (*p* < 0.0001) as compared to either 6-gingerol or metformin groups, respectively. In comparison with the control group, 6-gingerol and metformin groups showed significantly elevated levels of TLR4 (*p* < 0.0001), (*p* < 0.01); TRAF6 (*p* < 0.0001); NLRP3 (*p* < 0.0001); and caspase-1 (*p* < 0.0001). A non-significant difference was found between 6-gingerol + metformin group and control group regarding TLR4, NLRP3, and caspase-1 levels whereas TRAF6 level was significantly higher in 6-gingerol + metformin group compared to control group (*p* < 0.05).

### 6-Gingerol, metformin, and their combination decreased renal inflammatory cytokines levels and diminished the inflammatory response in DN

Since inflammatory cytokines are crucial for the onset and development of DN, protein levels of TNF-α and IL-1β were measured in kidney tissues. Figure 5E and F demonstrated a significant elevation in renal TNF-α and IL-1β levels in the DN group relative to the control group (*p* < 0.0001). A significant reduction of TNF-α level was observed in 6-gingerol, metformin, and 6-gingerol + metformin combination groups by 40.5%, 43.4%, and 52.6%, respectively, while IL-1β level was significantly decreased by 37.2%, 44.7%, and 63.1%, respectively. The 6-gingerol + metformin combination group exhibited a significantly reduced TNF-α level with regards to 6-gingerol (*p* < 0.001) and metformin (*p* < 0.01) groups. Also, IL-1β expressed a markedly reduced level in the 6-gingerol + metformin group in comparison with 6-gingerol and metformin groups (*p* < 0.0001). As compared to control group, 6-gingerol and metformin groups manifested significantly elevated TNF-α (*p* < 0.0001), (*p* < 0.001) and IL-1β (*p* < 0.0001) levels, respectively, whereas 6-gingerol + metformin group showed no significant difference as compared to control group.

### 6-Gingerol, Metformin, and their combination suppressed NF-κB (p65) protein expression in kidney tissue

**Fig. 6:**
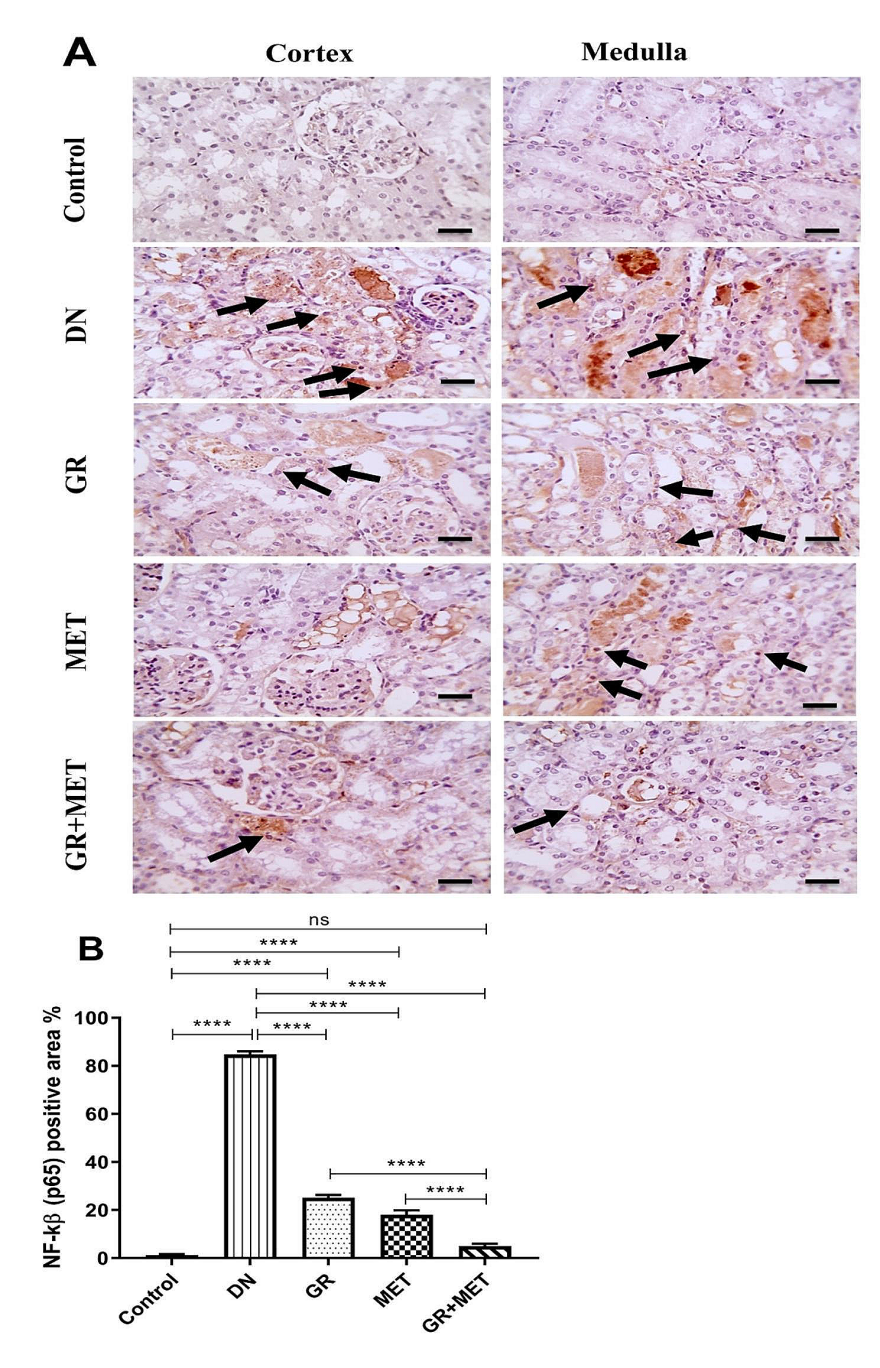
Immunohistochemical examination of NF-κB (p65) in renal tissue **A**: Microscopic images of immunostained renal sections against NF-κB (p65) showing negative staining in the cortex and medulla in the control group, excess brown tubular staining in the cortex and medulla in the DN group, decreased brown tubular staining in the cortex and medulla in GR group and MET group, and much more decreased brown tubular staining in the cortex and medulla in GR + MET group. Black arrows refer to nuclear staining. Immunohistochemistry is counterstained with Mayer’s hematoxylin. X: 400, scale bar = 50 micrometer **B**: Immunostaining of NF-κB (p65) was assessed quantitatively through the area of positive expression. DN: diabetic nephropathy, GR: 6-gingerol, MET: metformin, NF-κB (p65): nuclear factor-kappa B (p65). Data are represented as Mean $$ \pm $$ SEM (****p$$ <$$ 0.0001, ns: non-significant)

Protein expression of NF-κB (p65) in kidney tissue was examined by immunohistochemistry (Fig. 6A and B). Microscopic images of immunostained renal sections against NF-κB (p65) showed no brown staining in either the cortex or medulla in the control group. DN group showed excess brown tubular staining in both the cortex and medulla which implied an elevated expression of NF-κB (p65), as well as excess brown nuclear staining in tubules, which revealed the elevated nuclear expression of activated NF-κB (p65). NF-κB (p65) expression was semi-quantified by measuring NF-κB (p65) positive area percentage, which was significantly elevated in the DN group relative to the control group (*p* < 0.0001). Administration of 6-gingerol, metformin, or 6-gingerol + metformin combination to diabetic rats resulted in a significant reduction in NF-κB (p65) positive area percentage with regards to untreated diabetic rats (*p* < 0.0001). 6-Gingerol and metformin groups exhibited a significant elevation in NF-κB (p65) positive area percentage in comparison with control group (*p* < 0.0001). Moreover, the 6-gingerol + metformin group exhibited a significantly decreased NF-κB (p65) positive area percentage when compared with either 6-gingerol or metformin group (*p* < 0.0001) and a non-significant difference with respect to the control group.

### 6-Gingerol, Metformin, and their combination up-regulated miRNA-146a and miRNA-223 gene expression in diabetic kidneys

**Fig. 7:**
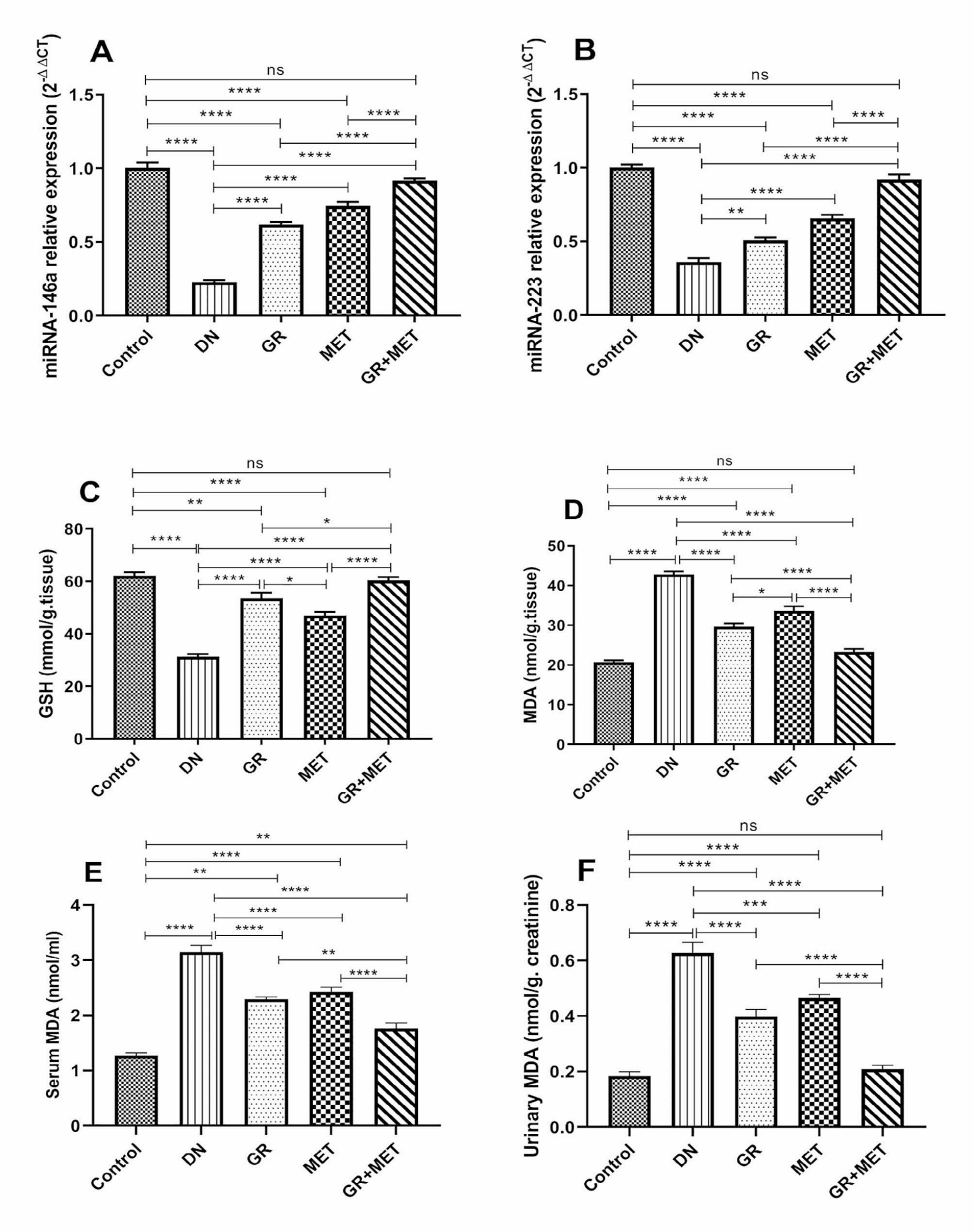
GR, MET, and their combination up-regulated miRNA-146a and miRNA-223, decreased oxidative stress and lipid peroxidation Relative expression of miRNA-146a (**A**) and miRNA-223 (**B**) was measured using quantitative real-time polymerase chain reaction (qRT-PCR). Oxidative stress was assessed by spectrophotometric measurement of the levels of reduced glutathione (GSH) (**C**) and malondialdehyde (MDA) (**D**) in renal tissue and the levels of serum MDA (**E**) and urinary MDA (**F**). DN: diabetic nephropathy, GR: 6-gingerol; MET: metformin. Data are represented as Mean $$ \pm $$ SEM (*p$$ <$$ 0.05, **p$$ <$$ 0.01, ***p$$ <$$ 0.001, ****p$$ <$$ 0.0001, ns: non-significant)

As shown in Fig. 7A and B, a significant down-regulation of miRNA-146a (4.4-fold) and miRNA-223 (2.8-fold) gene expression was observed in the DN group in comparison with the control group (*p* < 0.0001). When compared to the DN group, miRNA-146a gene expression was significantly higher in the 6-gingerol, metformin, and 6-gingerol + metformin groups (*p* < 0.0001). Also, miRNA-223 gene expression was significantly higher in 6-gingerol (*p* < 0.01), metformin (*p* < 0.0001), and 6-gingerol + metformin (*p* < 0.0001) groups with respect to the DN group. In addition, the 6-gingerol + metformin group revealed significantly up-regulated miRNA-146a and miRNA-223 gene expressions with respect to either the 6-gingerol or metformin group (*p* < 0.0001). Moreover, miRNA-146a and miRNA-223 were significantly decreased in 6-gingerol and metformin groups with respect to control group (*p* < 0.0001), while no significant difference was observed in 6-gingerol + metformin group with respect to control group.

### 6-Gingerol, Metformin, and their combination decreased oxidative stress in diabetic kidneys

Our results revealed a markedly improved oxidative status in diabetic rats that received 6-gingerol, metformin, or their combination. As shown in Fig. 7C, a significant reduction in renal GSH level was observed in the DN group relative to the control group (*p* < 0.0001) reflecting a state of high oxidative stress in diabetic rats. Administration of 6-gingerol, metformin, and 6-gingerol + metformin combination to diabetic rats brought about significantly elevated renal GSH levels by 71.2%, 50.2%, and 93%, respectively. Renal GSH level was markedly elevated in the 6-gingerol group relative to the metformin group (*p* < 0.05). Both 6-gingerol and metformin groups showed a significant decrease in renal GSH level (*p* < 0.01) and (*p* < 0.0001), respectively, compared to the control group. The 6-gingerol + metformin group revealed significantly elevated renal GSH levels when compared with 6-gingerol (*p* < 0.001) and metformin (*p* < 0.0001) groups and no significant difference relative to the control group.

Alternatively, Fig. 7D, E and F demonstrated significantly elevated MDA levels in renal tissue, serum and urine, respectively, in the DN group with respect to the control group (*p* < 0.0001) reflecting a significant increase in lipid peroxidation. Administration of 6-gingerol, metformin, and 6-gingerol + metformin combination to diabetic rats led to significantly reduced renal MDA levels by 30.8%, 21.4%, and 45.5%; serum MDA levels by 27.2%, 22.9%, and 44%; and urine MDA levels by 36.4%, 25.9%, and 66.7%, respectively. Renal MDA level was markedly lower in the 6-gingerol group relative to the metformin group (*p* < 0.05). The MDA levels were significantly reduced in renal (*p* < 0.0001), serum (*p* < 0.01), and urinary (*p* < 0.0001) samples in 6-gingerol + metformin group with respect to 6-gingerol group. The 6-gingerol + metformin group exhibited significantly lower renal, serum, and urinary MDA levels with respect to metformin group (*p* < 0.0001). Compared with control group, renal and urinary MDA levels were significantly higher in both 6-gingerol and metformin groups (*p* < 0.0001) and serum MDA level was significantly higher in 6-gingerol (*p* < 0.01) and metformin (*p* < 0.0001) groups. Besides, the 6-gingerol + metformin group displayed non-significantly different renal and urinary MDA levels and significantly higher serum MDA level (*p* < 0.01) when compared with the control group.

### 6-Gingerol, Metformin, and their combination suppressed renal fibrosis in diabetic kidneys

**Fig. 8:**
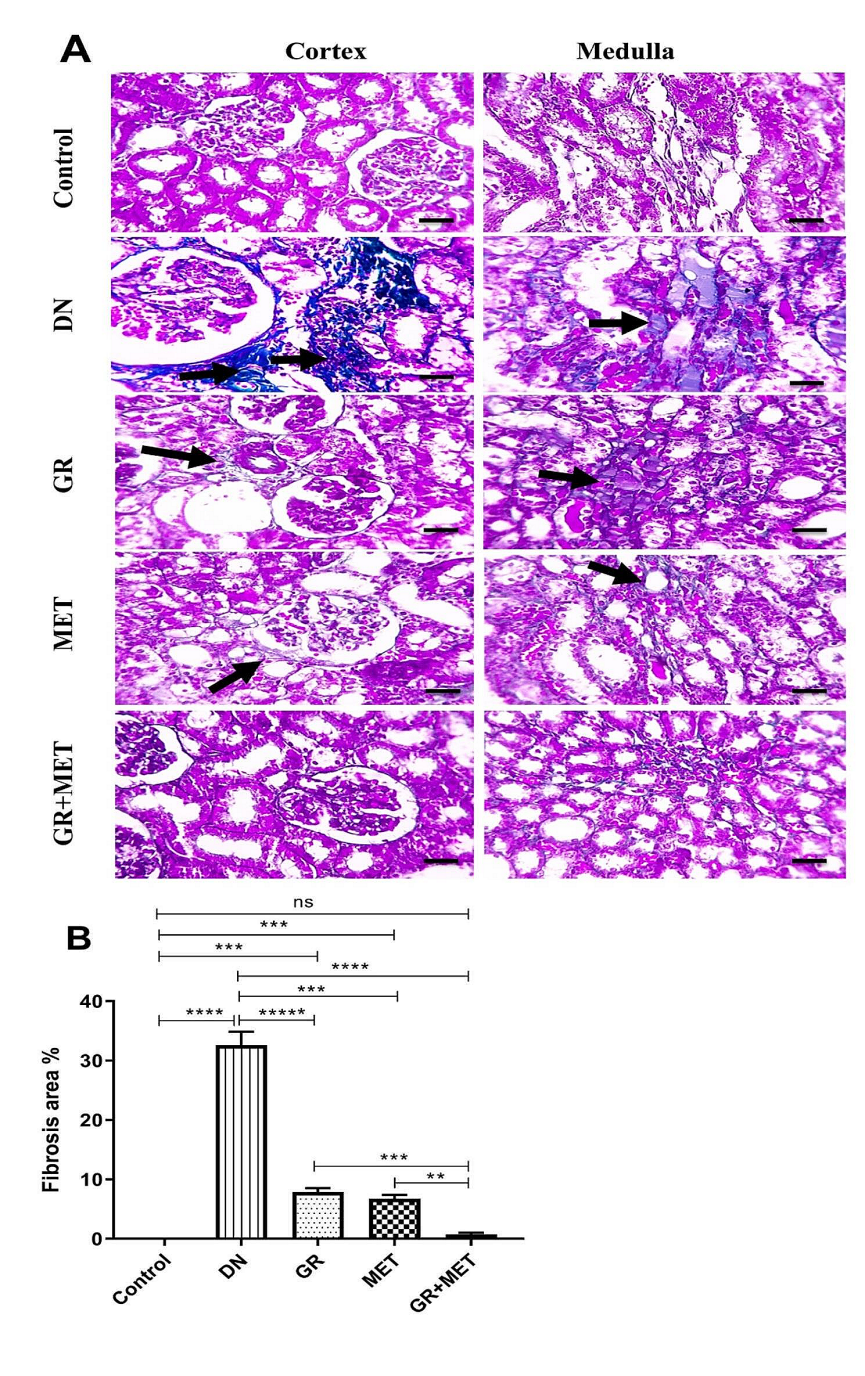
Histopathological examination of Masson’s trichrome-stained renal sections and fibrosis percentage assessment in diabetic rats **A**: Microscopic images of Masson’s trichrome-stained renal sections showing no collagen deposition in the cortex or medulla in the control group, excess bluish-stained collagen deposition in the cortex and medulla in the DN group, decreased collagen deposition in the cortex and medulla in the GR group and MET group, and much more decreased collagen deposition in the cortex and medulla in GR + MET group. Black arrows refer to collagen deposition. X: 400, scale bar = 50 micrometer **B**: Fibrosis percentage was assessed quantitatively through Masson’s-positive area percentage. DN: diabetic nephropathy; GR: 6-gingerol; MET: metformin. Data are represented as Mean $$ \pm $$ SEM (**p$$ <$$ 0.01, ***p$$ < $$0.001, ****p$$ <$$ 0.0001, ns: non-significant)

As demonstrated by Fig. 8A and B, Masson trichrome-stained renal tissue segments showed no excess collagen deposition in either the cortex or medulla in the control group. DN group showed excess bluish-stained collagen deposition in the renal cortex and medulla, reflecting significant fibrosis in the DN group. 6-Gingerol and metformin groups showed markedly less bluish-stained collagen deposition. The collagen deposition was even more significantly reduced in the 6-gingerol + metformin group. The fibrosis percentage was markedly decreased by 75.8% in the 6-gingerol group and by 79.3% in the metformin group relative to the DN group. 6-Gingerol and metformin groups exhibited a significant elevation in fibrosis percentage in comparison with control group (*p* < 0.001). In addition, the 6-gingerol + metformin group demonstrated a significantly decreased fibrosis percentage relative to either 6-gingerol (*p* < 0.001) or metformin (*p* < 0.01) groups and non-significant differences with respect to the control group.

**Fig. 9:**
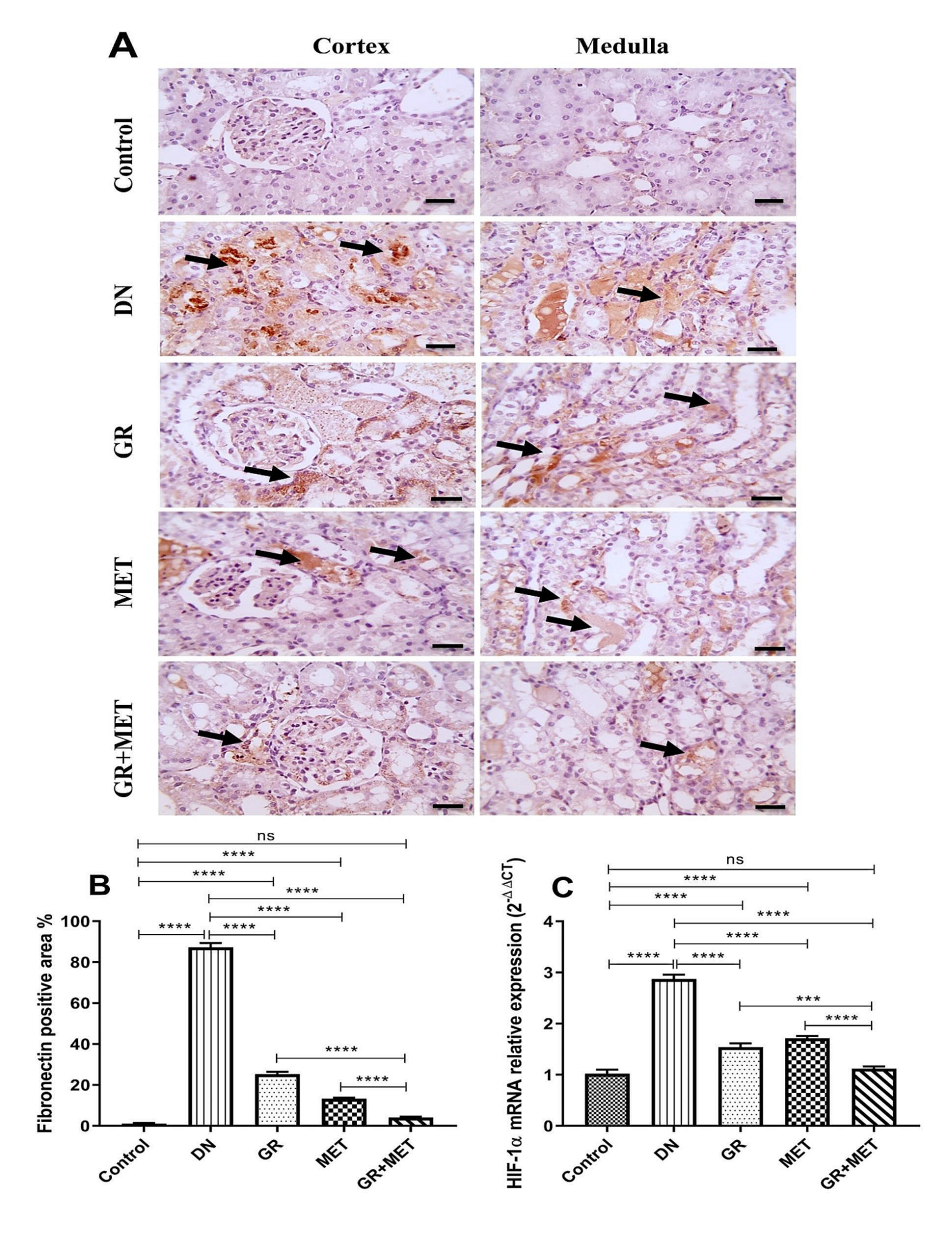
Immunohistochemical examination of fibronectin and gene expression of HIF-1α in renal tissue **A**: Microscopic images of immunostained renal sections against fibronectin showing negative staining in the cortex and medulla in the control group, excess brown tubular staining in the cortex and medulla in DN group, decreased brown tubular staining in the cortex and medulla in GR group and MET group, and much more decreased positive brown tubular staining in the cortex and medulla in GR + MET group. Black arrows refer to fibronectin deposition. Immunohistochemistry is counterstained with Mayer’s hematoxylin. X: 400, scale bar = 50 micrometer **B**: Immunostaining of fibronectin was assessed quantitatively through the area of positive expression. DN: diabetic nephropathy, GR: 6-gingerol, MET: metformin. Data are represented as Mean $$ \pm $$ SEM (****p$$ <$$ 0.0001) **C**: Renal hypoxia was assessed by measuring mRNA relative expression of hypoxia-inducible factor-1 alpha (HIF-1α). DN: diabetic nephropathy, GR: 6-gingerol, MET: metformin. Data are represented as Mean $$ \pm $$ SEM (***p$$ < $$0.001, ****p$$ <$$ 0.0001, ns: non-significant)

Fibronectin deposition in kidney tissue was stained immunohistochemically to further evaluate renal fibrosis (Fig. 9A and B). Microscopic images of immunostained kidney tissue segments against fibronectin showed no excess brown staining in the cortex and medulla in the control group. Meanwhile, excess brown tubular staining was expressed by the DN group in both the cortex and medulla, reflecting significantly increased fibronectin deposition relative to the control group (*p* < 0.0001). All treatment groups showed a remarkable reduction in brown tubular staining in both the cortex and medulla. 6-Gingerol and metformin groups demonstrated significantly decreased fibronectin deposition by 71.1% and 84.8%, respectively, in comparison with the DN group. However, 6-gingerol and metformin groups exhibited a significant elevation in fibronectin deposition in comparison with control group (*p* < 0.0001). The 6-gingerol + metformin group demonstrated significantly decreased fibronectin deposition when compared with 6-gingerol and metformin groups (*p* < 0.0001) and non-significantly different fibronectin deposition relative to the control group.

**Fig. 10:**
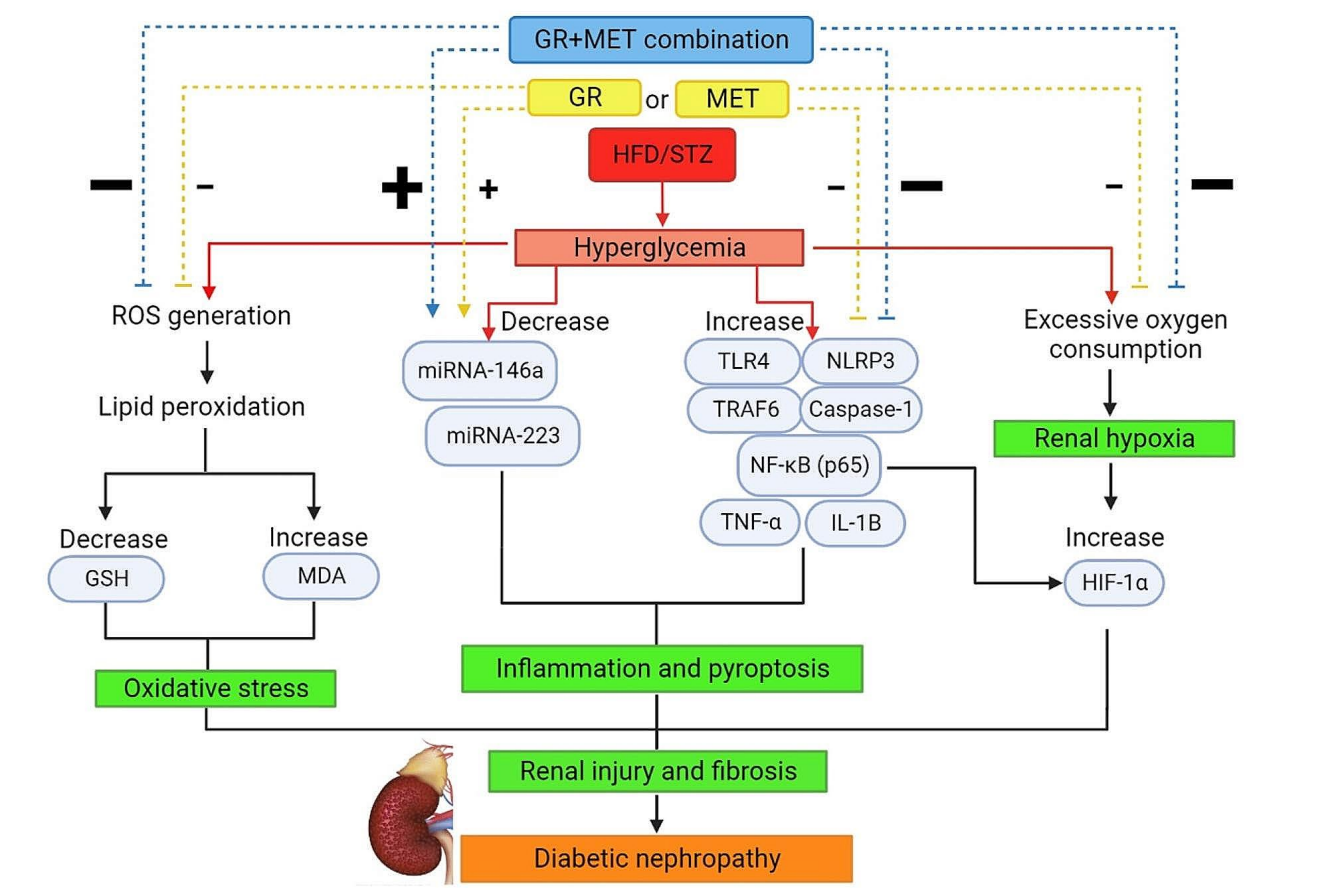
A summary figure of the mechanisms of action of GR, MET, and their combination in HFD/STZ-induced DN in rats DN: diabetic nephropathy; GR: 6-gingerol; MET: metformin; HFD: high-fat diet; STZ: streptozotocin; TLR4: Toll-like receptor 4; TRAF6: tumor necrosis factor receptor-associated Factor 6; NF-κB (p65): nuclear factor-kappa B (p65); NLRP3: NOD-like receptor family pyrin domain-containing 3; TNF-α: tumor necrosis factor-alpha; IL-1β: interleukin-1 beta; ROS: reactive oxygen species; GSH: reduced glutathione; MDA: malondialdehyde; HIF-1α: hypoxia-inducible factor-1 alpha.

### 6-Gingerol, Metformin, and their combination diminished renal hypoxia in DN

As shown in Fig. 8C, the DN group showed a 2.9-fold elevation in the mRNA expression of HIF-1α with respect to the control group (*p* < 0.0001). This elevation was significantly reduced when diabetic rats received 6-gingerol, metformin, or 6-gingerol + metformin combination by 46.4%, 40.4%, and 61%, respectively (*p* < 0.0001). However, 6-gingerol and metformin groups still exhibited a significant elevation in HIF-1α mRNA expression in comparison with control group (*p* < 0.0001). A marked reduction in HIF-1α mRNA expression was seen in the 6-gingerol + metformin group relative to 6-gingerol (*p* < 0.001) or metformin group (*p* < 0.0001) and a non-significant variation was found with respect to the control group.

### 6-Gingerol, Metformin, and their combination reduced fasting blood glucose and renal hypertrophy in DN

As shown in Table 2, DN group expressed a 4.2-fold elevated fasting blood glucose level relative to the control group (*p* < 0.0001). When diabetic rats were administered 6-gingerol, metformin, or 6-gingerol + metformin combination, fasting blood glucose was markedly lowered by 54.9%, 64.3%, and 74.2%, respectively. Noteworthy, fasting blood glucose was significantly lower in the 6-gingerol group relative to the metformin group (*p* < 0.05). 6-Gingerol + metformin combination group revealed a significantly decreased fasting blood glucose with regards to 6-gingerol (*p* < 0.0001) or metformin (*p* < 0.05) groups. Fasting blood glucose was still significantly elevated in 6-gingerol (*p* < 0.0001) and metformin (*p* < 0.01) groups relative to control group. Whereas the fasting blood glucose became normalized in rats receiving the 6-gingerol + metformin combination.

**Table 2:** 6-gingerol (GR), metformin (MET), and GR + MET effects on blood glucose, KW/BW index, and lipid profile

	Control	DN	GR	MET	GR + MET
**Fasting blood glucose (mg/dl)**	94.7 ± 4.1	395.4 ± 14.5^****^	178.3 ± 8.7^****, ####, $$$$, %^	141.2 ± 4.2^**, ####, $^	102.1 ± 4.6^####^
**Body weight (BW) (g)**	261.4 ± 6.2	145.2 ± 8.2^****^	189.6 ± 11.2^****, #, $^	195.3 ± 12.1^***, ##, $^	234.1 ± 7.9^####^
**Kidney weight (KW) (g)**	0.64 ± 0.02	0.89 ± 0.02^****^	0.72 ± 0.01^**,####, $$^	0.71 ± 0.01^####, $^	0.66 ± 0.01^####^
**KW/BW index (%)**	0.25 ± 0.01	0.63 ± 0.03^****^	0.39 ± 0.02^****, ####, $$^	0.37 ± 0.02 ^***, ####, $^	0.28 ± 0.01^####^
**Triglycerides (mg/dl)**	71.2 ± 1.4	184.1 ± 2.9^****^	84.6 ± 2.5^**, ####, $^	86.7 ± 2.6 ^***, ####, $$^	74.5 ± 1.2^####^
**Total cholesterol (mg/dl)**	72.6 ± 0.8	155.8 ± 3.2^****^	87.1 ± 1.8^***, ####, $$^	85.3 ± 1.9^***, ####, $^	76.9 ± 1.7^####^
**LDL cholesterol (mg/dl)**	18.5 ± 1.8	94.3 ± 2.3^****^	37.6 ± 1.9^****, ####, $$^	31.3 ± 1.7^***, ####^	25.9 ± 2.3^####^
**HDL cholesterol (mg/dl)**	41.1 ± 1.1	24.4 ± 1.3^****^	32.6 ± 2.0^**, ##^	36.9 ± 1.8^####^	36.1 ± 1.2^####^

High-fat diet/streptozotocin (HFD/STZ)-induced diabetic rats were treated daily for eight weeks with oral gavage of GR (100 mg/kg), MET (300 mg/kg), or their combination. Values are displayed in the form of means ± SEM (*n* = 8/group)

** represents a significant difference from the control group at *p* < 0.01.

*** represents a significant difference from the control group at *p* < 0.001

**** represents a significant difference from the control group at *p* < 0.0001

# represents a significant difference from the DN group at *p* < 0.05

## represents a significant difference from the DN group at *p* < 0.01

#### represents a significant difference from the DN group at *p* < 0.0001

$ represents a significant difference from the GR + MET group at *p* < 0.05

$$ represents a significant difference from the GR + MET group at *p* < 0.01

$$$$ represents a significant difference from the GR + MET group at *p* < 0.0001

% represents a significant difference from the MET group at *p* < 0.05

DN group showed a 32% increase in kidney weight/body weight (KW/BW) index relative to the control group (*p* < 0.0001), which reveals marked renal hypertrophy in diabetic rats (Table 2). Administration of 6-gingerol, metformin, and 6-gingerol + metformin combination to diabetic rats has significantly decreased the KW/BW index by 22.4%, 22.1%, and 32.4%, respectively. The 6-gingerol + metformin combination group exhibited a significantly decreased KW/BW index relative to 6-gingerol (*p* < 0.01) or metformin (*p* < 0.05) groups. KW/BW index was still significantly elevated in 6-gingerol (*p* < 0.0001) and metformin (*p* < 0.001) groups relative to control group whereas it was restored in diabetic rats treated with the 6-gingerol + metformin combination, i.e., no significant difference was found when compared to normal control rats.

### 6-Gingerol, Metformin, and their combination improved lipid profile in diabetic rats

Our results demonstrated an overall improvement in the lipid profile of diabetic rats receiving 6-gingerol, metformin, or their combination. As shown in Table 2, diabetic rats showed 2.6-fold, 2.1-fold, and 5.1-fold elevation in the levels of serum triglycerides, total cholesterol, and LDL cholesterol, respectively, as well as a 1.7-fold decrease in HDL cholesterol levels in comparison with control rats. 6-Gingerol, metformin, and 6-gingerol + metformin combination have significantly decreased triglycerides, total cholesterol, and LDL cholesterol (*p* < 0.0001) while significantly increased HDL cholesterol levels (*p* < 0.01), (*p* < 0.0001), and (*p* < 0.0001), respectively, with regards to the DN group. In addition, the 6-gingerol + metformin group revealed significantly decreased triglycerides (*p* < 0.05), (*p* < 0.01), and total cholesterol (*p* < 0.01), (*p* < 0.05) levels relative to 6-gingerol and metformin groups, respectively. The 6-gingerol + metformin group revealed significantly lower LDL cholesterol levels with regards to the 6-gingerol group (*p* < 0.01) and non-significantly lower LDL cholesterol levels with regards to the metformin group. No significant difference in HDL cholesterol existed in diabetic rats treated with the 6-gingerol + metformin combination relative to those treated with 6-gingerol or metformin. In comparison with the control group, 6-gingerol and metformin groups showed a significant elevation in serum triglycerides (*p* < 0.01) and (*p* < 0.001), total cholesterol (*p* < 0.001), and LDL cholesterol (*p* < 0.0001) and (*p* < 0.001), respectively. Regarding HDL cholesterol, a significant decrease was found in 6-gingerol group (*p* < 0.01) and a non-significant decrease was observed in metformin group, in comparison with the control group. Furthermore, no significant difference was observed between 6-gingerol + metformin group and control group regarding serum triglycerides, total cholesterol, LDL cholesterol and HDL cholesterol.

## Discussion

Diabetic nephropathy is a serious complication of diabetes mellitus that involves multiple hemodynamic and metabolic changes brought on by hyperglycemia, with inflammation and consequent fibrosis being the ultimate contributors to renal dysfunction [53]. The currently available oral anti-diabetic treatments have been insufficient to halt DN development and progression to end-stage renal disease [54]. This work aimed to investigate the proposed renoprotective effects of the natural compound 6-gingerol in DN and its underlying mechanisms of action. Also, the combination of 6-gingerol and the standard anti-hyperglycemic drug, metformin, was tested for potential premium renoprotective effects. To the best of our knowledge, this is the first study to identify the effect of 6-gingerol on renal expression of miRNA-146a and miRNA-223 as well as the downstream inflammatory pathway in the context of DN in rats. This study sheds light on the potential mechanisms underlying the renoprotective effects of 6-gingerol in DN. Additionally, our findings reveal the beneficial additive effect of combining 6-gingerol and metformin in DN, which has not been previously explored. However, further research is warranted to fully understand the underlying mechanisms and validate these findings in clinical settings.

The DN model was established experimentally in HFD/STZ-induced diabetic rats. High fat diet and a low dose of STZ were used to induce both peripheral insulin resistance as well as impairment of insulin production and secretion via partial degeneration of β-cells to mimic the natural pathophysiology of type 2 diabetes mellitus [43]. The success of DN induction in diabetic rats was confirmed by elevated urinary protein excretion levels in 24-hr urine samples (30.3 mg ± 1.4) which showed microalbuminuria [55]. Elevation of serum levels of creatinine and BUN, high proteinuria/creatininuria ratio and decline of creatinine clearance in diabetic rats as well as histopathological lesions observed in the renal cortex and medulla further confirmed the occurrence of renal injury. These results matched those of an earlier DN model study conducted in rats by Abou-Hany et al. [56].

When 6-gingerol was administered to diabetic rats, a considerable improvement in renal function tests and a remarkable enhancement in the renal glomerular and tubular structure were observed, which further confirms the renoprotective effect of 6-gingerol as previously reported by Almatroodi, et al. [41]. In addition, the combination of 6-gingerol and metformin attained much improved renal function tests and renal histological picture, which indicates a superior renoprotective effect for the combination group. This finding suggests that combining these medications may offer enhanced therapeutic benefits for the management of DN. Furthermore, it raises the possibility of reducing the dosage of metformin to minimize potential adverse effects while maintaining or even improving its efficacy.

Diabetic rats demonstrated marked hyperglycemia and a considerable elevation in the KW/BW index and renal hypertrophy, which are early signs of DN induction [57, 58]. Our study revealed that 6-gingerol has a remarkable glucose-reducing outcome in diabetic rats, which appeared to be consistent with the results of Singh, et al. [59] in diabetic mice. Moreover, 6-gingerol significantly decreased the KW/BW index. However, fasting blood glucose levels and KW/BW index were still significantly elevated in the 6-gingerol group relative to the control group. Yet, the 6-gingerol and metformin combination was capable of restoring fasting blood glucose and KW/BW index to normal levels and preventing renal hypertrophy.

Dyslipidemia is common in type 2 diabetes mellitus, as insulin resistance affects enzymes and pathways of glucose and lipid metabolism [60]. This study’s results showed that in diabetic rats, serum triglycerides, total cholesterol, and LDL cholesterol levels increased while HDL cholesterol levels decreased. This was correspondent with the findings of Lecamwasam et al. [61] in diabetics suffering from CKD. Treatment of diabetic rats with 6-gingerol brought on a remarkable improvement in lipid profile that seemed to agree with Wang, et al. results in HFD/STZ-induced prediabetic mice [62]. Besides, the 6-gingerol + metformin combination showed more considerable improvements in lipid profile biomarkers.

In this context, metformin is known to activate AMP-activated protein kinase which leads to improved glucose uptake, enhanced fatty acid oxidation, and inhibition of pathways associated with excessive energy consumption, such as the mechanistic target of rapamycin (mTOR) signaling [63, 64]. Studies suggest that 6-gingerol may contribute to glycemic control by reducing blood glucose level, enhancing insulin sensitivity, increasing glucose uptake, and improving glucose metabolism. A study by Samad, et al. showed that 6-gingerol potentiated glucagon-like peptide-1 mediated glucose-stimulated insulin secretion pathway in pancreatic β-cells and improved hyperglycemia in type 2 diabetic rats [65]. By regulating glucose homeostasis, 6-gingerol may indirectly mitigate the detrimental effects of hyperglycemia on renal function [41, 66]. Thus, the combination of 6-gingerol and metformin may have complementary effects in regulating insulin signaling pathways, leading to enhanced glucose utilization, improved lipid metabolism and better glycemic control.

Inflammation is a key contributor to DN development and progression. Activation of the inflammasome complex and subsequent pyroptosis, as well as elevation of inflammatory cytokine levels, are hallmarks of the inflammatory process [67]. Our results showed the ability of 6-gingerol to suppress inflammation and pyroptosis in diabetic kidneys and attenuate DN by inhibiting TLR4/TRAF6/NLRP3 inflammasome signaling. This was evident by the down-regulation of TLR4, TRAF6, NF-κB (p65), NLRP3, and caspase-1 mRNA and protein expression in addition to the TNF-α and IL-1β level reduction in the renal tissues of diabetic rats receiving 6-gingerol. These effects were even more pronounced when diabetic rats received a combination of 6-gingerol and metformin. Our results agreed with those of earlier studies in liver injury, myocardial fibrosis, and mastitis experimental models [38, 68, 69]. In addition, a study by Song, et al. [42] demonstrated that 6-gingerol improved the condition of renal tissue in diabetic rats by alteration of p38 mitogen activated protein kinase and NF-κB and inhibition of cyclooxygenase-2, prostaglandin E2 and proinflammatory cytokines.

Noteworthy, our study declared the involvement of miRNA-146a in the posttranscriptional regulation of TLR4 signaling in the kidneys of DN-affected rats. Under normal conditions, the expression of miRNA-146a is induced by NF-κB while miRNA-146a possesses a fine-tuning effect that prevents, via a feedback mechanism, the over-activation of NF-κB. This is achieved through the down-regulation of IRAK-1 and TRAF6, the direct target genes of miRNA-146a, downstream of TLR4 and upstream of NF-κB. However, miRNA-146a is down-regulated in diabetic kidneys, which induces an up-regulation of NF-κB and various inflammatory cytokines and subsequent augmented inflammation [70, 71]. Our findings revealed the ability of 6-gingerol to up-regulate miRNA-146a expression in diabetic kidneys. This could explain its ability to inhibit TRAF6 and, consequently, NF-κB (p65) expression with subsequent inhibition of the inflammatory cytokines TNF-α and IL-1β.

It is worth mentioning that our findings showed that 6-gingerol treatment significantly reduced TLR4 expression, which could be partly explained by miRNA-146a’s ability to suppress TLR4 expression. In agreement with our results, miR-146a was shown by He, et al. [72] and Liu, et al. [73] to negatively regulate TLR4 expression in an ovarian dysfunction mouse model and fibroblast-like synoviocytes in rheumatoid arthritis patients, respectively. Contrarily, Morishita, et al. [74] and Petrkova, et al. [75] demonstrated that TLR4 was not suppressed by miRNA-146a expression in unilateral ureteral obstruction-induced renal fibrosis in mice and in patients with aortic valve stenosis, respectively. This might partly reflect a sort of specific localization and will require further investigations to find out whether TLR4 could be a direct target of miRNA-146a in diabetic kidneys and whether 6-gingerol could inhibit TLR4 expression via miRNA-146a up-regulation or a different mechanism.

MiRNA-223 proved to be a direct epigenetic regulator of NLRP3 inflammasome expression in different experimental models such as calcium oxalate-induced renal inflammation [76] and gouty inflammation [77]. Our study revealed a significant down-regulation of renal miRNA-223 expression in rats with DN. These results agreed with those obtained by Yu, et al. [78] in HBV-transfected podocytes and by Xu, et al. [79] in diabetic cardiomyopathy. In our research, treating diabetic rats with 6-gingerol led to a significant induction of miRNA-223 expression, which brought about a remarkable decline in the levels of NLRP3, caspase-1, and IL-1β. Noteworthy, the 6-gingerol and metformin combination produced much more considerable up-regulation of both miRNA-146a and miRNA-223 expression.

The specific molecular mechanisms underlying the cooperative effects of 6-gingerol and metformin in mitigating DN remain to be fully elucidated. In this context, both 6-gingerol and metformin have been shown to modulate the activity of transcription factors that directly regulate miRNA-146a and miRNA-223 expression, the epigenetic regulators of the TLR4/TRAF6/NLRP3 inflammasome pathway. For instance, 6-gingerol has been reported to activate nuclear factor erythroid 2-related factor 2 [37], while metformin can activate peroxisome proliferator-activated receptor gamma [80]. By independently activating these common transcription factors, 6-gingerol and metformin may additively enhance the expression of miRNA-146a and miRNA-223 [81, 82]. Furthermore, 6-gingerol and metformin possess complementary anti-inflammatory properties and can modulate inflammatory signaling pathways, which are closely linked to miRNA regulation. Both compounds have been shown to inhibit NF-κB signaling, which plays a key role in regulation of miRNA-146a expression [83, 84]. Additionally, they can suppress pro-inflammatory cytokines, such as interleukin-6 [42, 85], which can indirectly influence miRNA-223 expression [86]. Thus, 6-gingerol and metformin may cooperatively induce the expression of miRNA-146a and miRNA-223 and thus additively inhibit TLR4/TRAF6/NLRP3 inflammasome signaling and subsequent renal damage.

Diabetes and its complications are greatly influenced by oxidative stress. Hyperglycemia induces excessive ROS production, resulting in an imbalance between free radicals and antioxidant defense mechanisms. Lipid peroxidation occurs as a result of the interactions between ROS and polyunsaturated fatty acids, which plays an important role in diabetes-associated complications, including renal injury [87–90]. Our results revealed a high oxidative status in diabetic rats. This was obvious through a considerable reduction in the antioxidant GSH level in renal tissue and a remarkable elevation in the reactive aldehyde MDA levels in the renal tissue, serum and urine. Both Abou-Hany, et al. [56] and Mi, et al. [91] reported comparable outcomes in DN models. 6-Gingerol treatment of diabetic rats increased GSH levels, reduced MDA levels, and alleviated oxidative stress, possibly due to its well-recognized antioxidant capacity [38, 92].

Additionally, the antioxidant potential of 6-gingerol was further significantly enhanced when metformin was used in combination with 6-gingerol. The combination of 6-gingerol and metformin seemed to have a complementary effect in reducing oxidative stress and protecting cells from oxidative damage owing to the antioxidant properties of both compounds. Metformin can reduce oxidative stress by inhibiting ROS production through AMP-activated protein kinase activation, mitochondrial complex I inhibition, and increased antioxidant enzyme activity [63, 93]. Whereas 6-gingerol exhibits antioxidant activity via ROS scavenging, nuclear factor erythroid 2-related factor 2 up-regulation, improving the activities of antioxidant enzymes catalase, superoxide dismutase, glutathione peroxidase, and glutathione S-transferase as well as GSH level and reducing the level of MDA [37, 41, 94, 95]. In addition to these mechanisms, other studies have reported further evidence supporting the renoprotective effects of 6-gingerol in different renal injury models. A study by Tahoun, et al. showed the ability of 6-gingerol to inhibit inflammation, oxidative stress and apoptosis in cisplatin-induced nephrotoxicity via reduction of IL-1β, TNF-α, interleukin-6, inducible nitric oxide synthase, nitric oxide, MDA and caspase-3 in renal tissue [96]. Also, 6-gingerol was found to protect against gentamicin-induced renal cortex apoptosis and oxidative stress in rats via inhibition of caspase-3 and anti-heat shock protein 47 [97].

Renal fibrosis is an important pathological feature of diabetic kidneys and represents the final common pathway in the progression of DN to end-stage renal disease. Hyperglycemia-induced metabolic alterations trigger a state of chronic inflammation, which causes persistent injury in diabetic kidneys. This promotes various pathological changes, including epithelial-to-mesenchymal transition, endothelial-to-mesenchymal transition, and activation of fibroblasts and pericytes. These pathological changes cause the extracellular matrix components collagen and fibronectin to be deposited in excess, resulting in kidney fibrosis [98, 99].

Hypoxia is an important driving factor for DN and CKD. Excessive oxygen consumption brought on by diabetes-related metabolic changes causes renal tissue hypoxia and increased expression of HIF-1α; the crucial transcriptional regulator of cellular accommodation with hypoxia [100]. A major crosstalk was found between hypoxia and inflammation. Previous studies showed that activation of NF-κB increased the expression of HIF-1α. Furthermore, HIF-1α was required for hypoxia-promoted TLR4 expression and downstream NF-κB transcriptional activation as well [19, 101–103]. In addition, previous studies have shown that increased expression of HIF-1α in diabetic kidneys contributed to renal fibrosis and the progression of DN [21, 104, 105].

The results of our study revealed significant renal fibrosis in untreated diabetic rats, evidenced by the significantly increased collagen fibril deposition in Masson-stained kidney tissue segments. This was further confirmed by the elevated expression of the fibrosis hallmark protein, fibronectin, in both cortex and medulla of immunostained renal tissue from diabetic rats. Additionally, our study revealed a significant elevation of renal HIF-1α expression in diabetic rats. These results were in line with those recorded both in vitro and in vivo by Mei, et al. [105], who discovered that elevated HIF-1α expression and susceptibility to fibrosis in diabetes are significantly correlated.

As indicated by our results, 6-gingerol significantly ameliorated renal fibrosis in diabetic rats possibly as a result of its anti-inflammatory and antioxidant capabilities. Also, 6-gingerol was able to reduce renal hypoxia and HIF-1α expression significantly, which could contribute to its anti-fibrotic effect. Moreover, the anti-fibrotic as well as anti-hypoxic capacities of 6-gingerol were further significantly enhanced when metformin was used in combination with 6-gingerol suggesting that 6-gingerol and metformin may have complementary effects in mitigating hypoxia and fibrosis in DN. 6-Gingerol has been reported to suppress the expression of HIF-1α and reduce hypoxia in lung cancer [106]. 6-Gingerol has been shown to inhibit renal fibrosis through various mechanisms, including the suppression of transforming growth factor-beta 1 signaling and the inhibition of fibroblast activation and extracellular matrix deposition [42]. Metformin treatment was reported to relieve the processes of inflammation and fibrosis in individuals with diabetic kidney disease by reducing the levels of the Tenascin-C, p-NF-κB (p65), connective tissue growth factor, and fibronectin proteins [83]. Together, these actions may contribute to the reversal of hypoxia and fibrosis in the kidney.

Overall, the findings of this study suggest that 6-gingerol is promising for the prevention of DN. Our study showed that 6-gingerol exerted a significant renoprotective effect through multiple mechanisms including inhibition of inflammation via modulation of miRNA-146a, miRNA-223 and TLR4/NF-κB/NLRP3 inflammasome pathway, reduction of oxidative stress, hypoxia, and fibrosis as well as glycemic control. Being a natural compound, the renoprotective effects of 6-gingerol, as demonstrated in the study, make it an attractive option for patients seeking natural alternative therapies or complementary therapies alongside conventional pharmacological treatments. The combination therapy of 6-gingerol and metformin provides superior renoprotection compared to monotherapy which demonstrates significant potential for clinical application. The combination therapy has the advantage of addressing multiple mechanisms involved in the development and progression of DN by targeting both metabolic and renal-related pathways. This multi-mechanistic strategy has the potential to yield improved outcomes in the management of DN. Another advantage of the combination therapy is the potential to reduce the dosage of metformin while maintaining or enhancing its effectiveness. By lowering the metformin dosage, the adverse effects associated with it, such as gastrointestinal symptoms, may be minimized, leading to better tolerability and adherence to treatment among DN patients.

However, it’s important to note that the study was conducted in an animal model of DN. Further research, particularly in the form of clinical trials involving human subjects, is essential to validate these findings and assess their applicability to clinical practice. Replicating the results in clinical studies would provide stronger evidence for considering the combination therapy of 6-gingerol and metformin in treatment guidelines and clinical practice guidelines for DN. While the findings of the study are promising, the translation of these findings into clinical practice requires careful evaluation of safety, efficacy, optimal dosing, long term benefits, potential drug interactions, and individual patient factors.

Future studies should focus on exploring different dosages and dosing regimens of 6-gingerol and metformin to determine the optimal therapeutic dose that achieves the highest efficacy and safety. Conducting long-term studies would provide valuable insights into the long-term effects and safety profile of the combination therapy. Further mechanistic studies should be performed to deepen our understanding of the underlying mechanisms by which 6-gingerol and metformin confer renoprotective effects. Clinical trials involving human subjects could be conducted to assess the impact of the combination therapy on renal function, glycemic control, inflammation, oxidative stress, and overall patient outcomes. Comparative studies comparing the combination therapy of 6-gingerol and metformin with other existing standard treatments or emerging therapies for DN could be performed which could help guide treatment decisions and determine the feasibility of 6-gingerol and metformin combination therapy in the management of DN.

## Conclusion

This study disclosed that 6-gingerol is a promising natural compound for the prevention of DN. Inhibition of inflammation, oxidative stress, hypoxia, and fibrosis are the key mechanisms in 6-gingerol’s renoprotective effect, which proved to be related to the induction of renal expression of miRNA-146a and miRNA-223 and subsequent inhibition of the TLR4/TRAF6/NLRP3 inflammasome pathway. The renoprotective effect of 6-gingerol appeared to be comparable to that of the standard anti-hyperglycemic drug, metformin, in HFD/STZ-induced diabetic rats. Moreover, the 6-gingerol and metformin combination revealed superior renoprotection compared to the use of each drug alone which suggests the potential to lower the dosage of metformin to minimize adverse effects while still maintaining or even improving its effectiveness (Fig. 10). However, further research is needed to determine the optimal dosage and evaluate the balance between efficacy and safety when using reduced doses of metformin in combination with 6-gingerol. Future studies should focus on elucidating further underlying mechanisms and conducting clinical trials to evaluate the safety and effectiveness of this drug combination in patients with DN.

## Data Availability

Data will be made available from the corresponding author upon reasonable request.
